# 79. Shotgun Metagenomics Reveals Microbial Ecological Features Driving Fecal Microbiota Transplantation Success in Carbapenem-Resistant Enterobacteriaceae Carriers

**DOI:** 10.1093/ofid/ofaf695.030

**Published:** 2026-01-11

**Authors:** Imchang Lee, Ki Tae Suk, Bong-Soo Kim, Seung Soon Lee

**Affiliations:** Hallym University Chuncheon Sacred Heart Hospital, Chuncheon-Si, Kangwon-do, Republic of Korea; Chuncheon Sacred Heart Hospital, Hallym University College of Medicine, Chuncheon-si, Kangwon-do, Republic of Korea; Ewha Womans University, Seoul, Seoul-t'ukpyolsi, Republic of Korea; Hallym University Chuncheon Sacred Heart Hospital, Hallym University College of Medicine, Chuncheon, Kangwon-do, Republic of Korea

## Abstract

**Background:**

Fecal microbiota transplantation (FMT) is a promising intervention for decolonizing carbapenem-resistant Enterobacteriaceae (CRE); however, predictors of success remain poorly understood. We aimed to identify pre-FMT microbial ecological features, and post-FMT microbiome dynamics associated with FMT efficacy using shotgun metagenomic sequencing.

**Figure:**
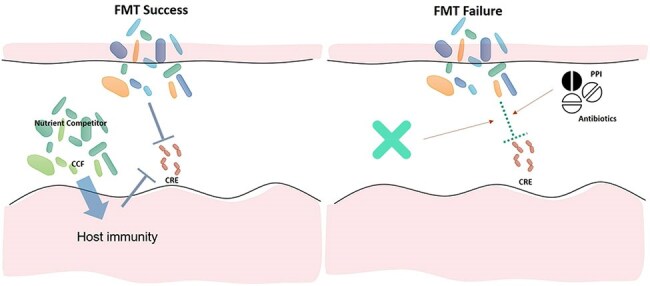

**Methods:**

FMT was performed in 21 patients colonized with CRE, using stool from 13 rigorously screened healthy donors. Longitudinal stool samples, collected from pre-FMT through 5 weeks post-FMT, underwent shotgun metagenomic sequencing. Patients were classified as responders (n = 9) or non-responders (n = 12) based on CRE clearance. We assessed taxonomic composition (phylum to species level), alpha diversity, dysbiosis scores, and donor–recipient similarity. Clinical metadata, including post-FMT exposure to antibiotics and non-antibiotic drugs (e.g., proton pump inhibitors [PPIs]), were also evaluated.

**Results:**

Responders showed a sustained decrease in CRE abundance along with a marked expansion of Actinomycetota and Bacteroidota. They also demonstrated significant recovery of alpha diversity and reductions in dysbiosis scores with non-responders. Compositional shifts in responders aligned closely with donor profiles, whereas non-responders diverged. This convergence of microbiota in responders was quantified by increasing donor–recipient similarity over time. Pre-FMT microbiota enriched with nutrient competitors, together with post-FMT shifts favoring colonization-permissive taxa (e.g., *Bacteroides* species), correlated with improved outcomes. In contrast, post-FMT exposure to antibiotics and PPIs was associated with decolonization failure at 1 month after FMT.

**Conclusion:**

FMT success in CRE decolonization is facilitated by ecological permissiveness—specifically, the pre-FMT presence of nutrient competitors, along with post-FMT shifts favoring colonization-permissive taxa and with alignment to donor microbiota. These dynamics were modulated by post-FMT exposure to drugs such as antibiotics and PPIs. Our findings suggest that microbiome-informed recipient profiling and rigorous post-FMT antibiotic and PPI stewardship may enhance the efficacy of FMT for CRE decolonization.

**Disclosures:**

All Authors: No reported disclosures

